# First-in-human evaluation of a no-alpha interleukin–2 mutein: safety and preliminary pharmacodynamic and clinical effect

**DOI:** 10.3389/fimmu.2025.1589042

**Published:** 2025-05-23

**Authors:** Iraida Caballero Aguirrechu, Braulio Mestre Fernández, Jorge Luis Soriano García, Nora Lim Alonso, Andrés Soto García, Vilma Fleites Calvo, Daines Mariño de la Puente, Geidy Vega Carvajal, Jenny Carolina Ávila Pérez, Ivis Mendoza Hernández, Elena García López, Alicia Tarinas Reyes, Gisela García-Pérez, Claudia Díaz Borges, Nuris Ledón Naranjo, Sum Lai Lozada Chang, Yanelda García Vega, Alexis Alvárez Lobaina, Mabel Alvárez Cardona, Patricia Lorenzo-Luaces Alvárez, Tania Crombet Ramos, Tania Carmenate Portilla, Kalet León Monzón

**Affiliations:** ^1^ Hermanos Ameijeiras Hospital, Oncology Department, Havana, Cuba; ^2^ National Institute of Oncology and Radiobiology, Havana, Cuba; ^3^ National Coordinating Center of Clinical Trials, Havana, Cuba; ^4^ Center of Molecular Immunology, Havana, Cuba

**Keywords:** interleukin-2, mutein, first-in-human, phase I, cancer immunotherapy

## Abstract

**Introduction:**

Interleukin 2 (IL-2) is essential for immune system activation. To reduce toxicity and prevent the activation of regulatory T cells (T-regs), a novel IL-2 variant containing 4-point mutations that prevent its interaction with the alpha chain of the receptor was designed. In preclinical studies, the no-alpha mutein preferentially stimulate CD8-T cells and natural killer (NK) cells compared to Tregs. Mutein also showed greater antitumor capacity than the native molecule in several tumor models.

**Methods:**

Patients with advanced solid tumors were included in a single-arm dose-escalation Phase I trial. The objectives of this study were to evaluate the safety and identify the recommended phase 2 dose. The effects on the most important immune subpopulations and preliminary objective response were also assessed. The protocol was listed in the National Registry for Clinical Trials (https://rpcec.sld.cu/ensayos/RPCEC00000234-En).

**Results and Discussion:**

In this phase I trial, 13 patients with advanced cancer were treated with five dose levels of IL-2 mutein, from 300 to 2400 IU/kg. The treatment was safe, and the maximum tolerated dose was not reached. Dose escalation did not continue, as a greater clinical and pharmacodynamic effect was observed at intermediate doses. One patient developed a possibly related serious event consisting on ventricular dysfunction and pneumonitis. No toxic deaths or vascular leak syndromes were detected, and the most frequent toxicities were chills, fever, and tachycardia. After treatment, most patients experienced an expansion of the total lymphocyte counts and the CD8-T cells and NK cells.

**Clinical trial registration:**

https://rpcec.sld.cu/ensayos/RPCEC00000234-En, identifier RPCEC00000234.

## Introduction

Immunotherapy is one of the most important pillars in the management of malignant tumors (1). The administration of Coley’s toxin in 1891 (2), the use of BCG for treating bladder cancer since 1990 (3), the approval of sipuleucel T for castration-resistant prostate cancer patients in 2010 (4), and the registration of anti-CTLA4 (5), anti-PD1 (6) and anti-LAG-3 antibodies (7) for metastatic melanoma in 2011, 2014, and 2022, respectively, represent some historical milestones of cancer immunotherapy. Antibodies against immune checkpoints are currently used for treating more than 20 tumor types in advanced, adjuvant, neoadjuvant, and perioperative scenarios (8). Other major immunotherapy achievements have been therapies based on adoptive cell transfer, including chimeric antigen receptor (CAR) T cells, recommended since 2017 for controlling hematological malignancies (9) as well as the use of tumor-infiltrating lymphocytes (10) approved since 2024 for metastatic melanoma patients.

In 1992, interleukin 2 (IL-2) was registered as a treatment for renal cell carcinoma patients (11). Six years later, it was accepted as a new alternative treatment for patients with metastatic melanoma (12). Despite very positive results, especially in terms of the duration of response for patients achieving complete or partial response, its use has been limited by severe toxicities and by the contraction of the immune effector response, following the activation of regulatory T cells (T-regs) (13).

Interleukin 2 is a pleiotropic cytokine that is essential for the activation of the immune system. This cytokine exerts its biological function by activating the interleukin 2 receptor, which can exist as a dimeric complex with beta (CD122) and gamma (CD132) chains or as a trimeric cluster containing alpha (CD25), beta and gamma chains (14). The beta and gamma chains conformed the IL-2 receptor of intermediate affinity (KD=10^-9^M) (15). This two-chains complex is expressed primarily by natural killer (NK) cells, as well as by CD4 and CD8 lymphocytes with a memory phenotype (14). In contrast, the trimeric receptor has a higher affinity (KD=10^-11^M) (15) and is constitutively expressed by regulatory T cells as well as by CD4 and CD8 T lymphocytes after antigenic stimulation (14).

Interleukin 2 can also act on other cells that are not part of the immune system, including vascular epithelial cells (16). The vascular leak syndrome, one of the most severe IL-2 associated toxicity, has been attributed to the interaction of the cytokine with the trimeric receptor in endothelial cells (17, 18).

To reduce IL-2 toxicity and prevent the activation of regulatory T cells, the Center for Molecular Immunology (Havana, Cuba) developed a variant with 4-point mutations which prevented its interaction with the alpha chain of the IL-2 receptor (CD25) (19).

In preclinical *in vivo* studies, the no-alpha mutein demonstrated its ability to preferentially stimulate CD8+ T cells and NK cells compared to T-regs with a CD4+CD25+FoxP3+ phenotype. In experimental tumor mouse models of lung, breast, colon and metastatic melanoma, the mutated variant showed greater antitumor capacity than the native molecule (19). The mutein was safer than the wild-type IL-2 (20).

The present clinical trial was designed as a first in human study to assess the safety, pharmacodynamics and preliminary effects of the no-alpha IL-2 mutein in patients with advanced solid tumors.

## Materials and methods

A single-arm dose-escalation Phase I/II clinical trial was designed to evaluate the safety and preliminary effects of the no-alpha IL-2 mutein. Patients of both sexes, older than 18 years, with any solid tumors showing progressive disease and no further treatment alternatives, were eligible for the trial. Other inclusion criteria were as follows: ECOG performance status ≤ 2, life expectancy of at least 3 months, organ and bone marrow function defined by the following parameters: hemoglobin ≥ 10 g/dl, total leukocyte count ≥ 3 x 10 ^9/^L, platelet count ≥ 100 x 10 ^9/^L, total bilirubin and creatinine within normal limits for the institution, and alanine amino transferase (ALT) or aspartate amino transferase (AST) ≤ 2.5 times the institutional upper normal limit. The most important exclusion criteria were pregnancy or breastfeeding, acute allergic states or history of severe allergic reactions, acute or chronic decompensated lung diseases that could interfere with the monitoring of the underlying disease, and previous history of demyelinating or inflammatory diseases of the central or peripheral nervous system. Other exclusion criteria were the existence of uncontrolled intercurrent illnesses including, but not limited to, active infections, symptomatic congestive heart failure, unstable chest angina, cardiac arrhythmia, diabetes mellitus, psychiatric illnesses, and brain metastases.

The main objectives of this study were to evaluate the safety of the IL-2 mutein and identify the recommended phase 2 dose (RP2D). Other objectives included the evaluation of the effect of IL-2 mutein on the most important immune subpopulations and the assessment of the preliminary objective response and survival of the treated patients. The second part of the clinical trial (phase II) using the RP2D, is currently ongoing.

Patients were admitted to the intensive care unit (ICU) for treatment administration, considering the potential side effects of the native interleukin-2. The non-alpha IL-2 mutein was administered intravenously (diluted in dextrose), via a central venous catheter, over 15 to 30 minutes, one dose every 8 hours until completing 14 doses per cycle (4.6 days or 110 hours). Patients then had a rest period of 9 days, after which they received the second cycle, using the same dose and scheme.

A continuous reassignment method was used for dose escalation, and four dose levels were initially established: 300, 600, 900, and 1200 IU/kg. According to the original design, the dose could be further escalated to a superior level if there is no evidence of dose-limiting toxicity (DLT). Patients were allocated to the next dose level when the preceding subject had completed the first 14 doses of treatment without signs or symptoms that precluded treatment continuation. Excluding safety interruptions, patients who completed at least 9 doses of treatment (70% of the planned dose) were considered evaluable. If a patient could not complete at least nine administrations of IL-2 mutein, another subject was included in the same cohort. Enrollment of patients at successive dose levels continued in an ascending manner (one patient per dose level) under the same criteria. If a patient developed DLT, 2 additional subjects were included in the same cohort. If no DLT occurred, the dose was escalated to the next level. Alternatively, if DLT was found, the dose was de-escalated based on the dose-toxicity model updated with the accumulated information. Dose evaluation was continued until 6 patients were treated at the same level. Adverse events (AE) were classified according to the Common Terminology Criteria for Adverse Events (CTCAE) version 5.0 of the United States National Cancer Institute. The occurrence of a definitively, probably, or possibly related serious adverse event with intensity ≥ 4 was defined as DLT.

The response to treatment was assessed using the Response Evaluation Criteria in Solid Tumors (RECIST) version 1.1, 4 weeks after the last dose of the second cycle and at months 6, 9, and 12. Disease control rate (DCR) was defined as the proportion of patients who achieved complete response, partial response or disease stabilization. Follow-up was maintained until patient death or until completion of one-year follow-up.

Waterfall and swimmer plots were used to illustrate preliminary clinical effects on tumor response and survival, respectively.

Data were analyzed in two settings: intention-to-treat (ITT) and per-protocol (PP). The PP population was defined as those patients who met all inclusion criteria and none of the exclusion criteria that received treatment as indicated in the protocol (no less than nine doses of the no-alpha IL2 mutein), in whom at least one assessment of the primary efficacy variable (objective response) was available. Survival was calculated by estimating the time elapsed from patient inclusion in the study until death or the latest news.

For the immune response evaluation, blood samples were collected on day 0, 24 hours after each cycle and 1 month after treatment completion. Peripheral blood mononuclear cells (PBMC) were isolated and cell suspensions were stained with the following fluorochrome-conjugated, anti-human antibodies: CD3 PE (HI3a), CD8 PECy7 (SK1), CD56 FITC (TULY56), Ki67 APC (20RAJ1), Foxp3 PE (236A/E7), CD4 FITC (OKT-4), all from BD Biosciences, and CD25 PEcy7 (BC96) from BioLegend. Briefly, cells were incubated with antibodies for 30 min in an ice bath and then washed. For intracellular staining, the Foxp3 staining buffer system (eBioscience) was used. Samples were acquired using a Gallios cytometer (Beckman Coulter) and analyzed using the Kaluza software. For statistical analysis, the paired T-test was applied, using the Graphpad 9.3.1 software.

This clinical trial was conducted in accordance with Guideline 6 of the International Conference on Harmonization (ICH), the Good Clinical Practices´ standard operating procedures of the Center of Molecular Immunology and the National Coordinating Center of Clinical Trials. The study was performed in accordance with the principles of the Declaration of Helsinki. The protocol was approved by the ethical committees of the two participating institutions, the National Institute of Oncology and Radiobiology and the Hermanos Ameijeiras Hospital, before enrolling the first patient. The protocol was listed in the National Registry for Clinical Trials (https://rpcec.sld.cu/ensayos/RPCEC00000234-En). All the patients signed an informed consent form.

## Results

**Figure 1:**
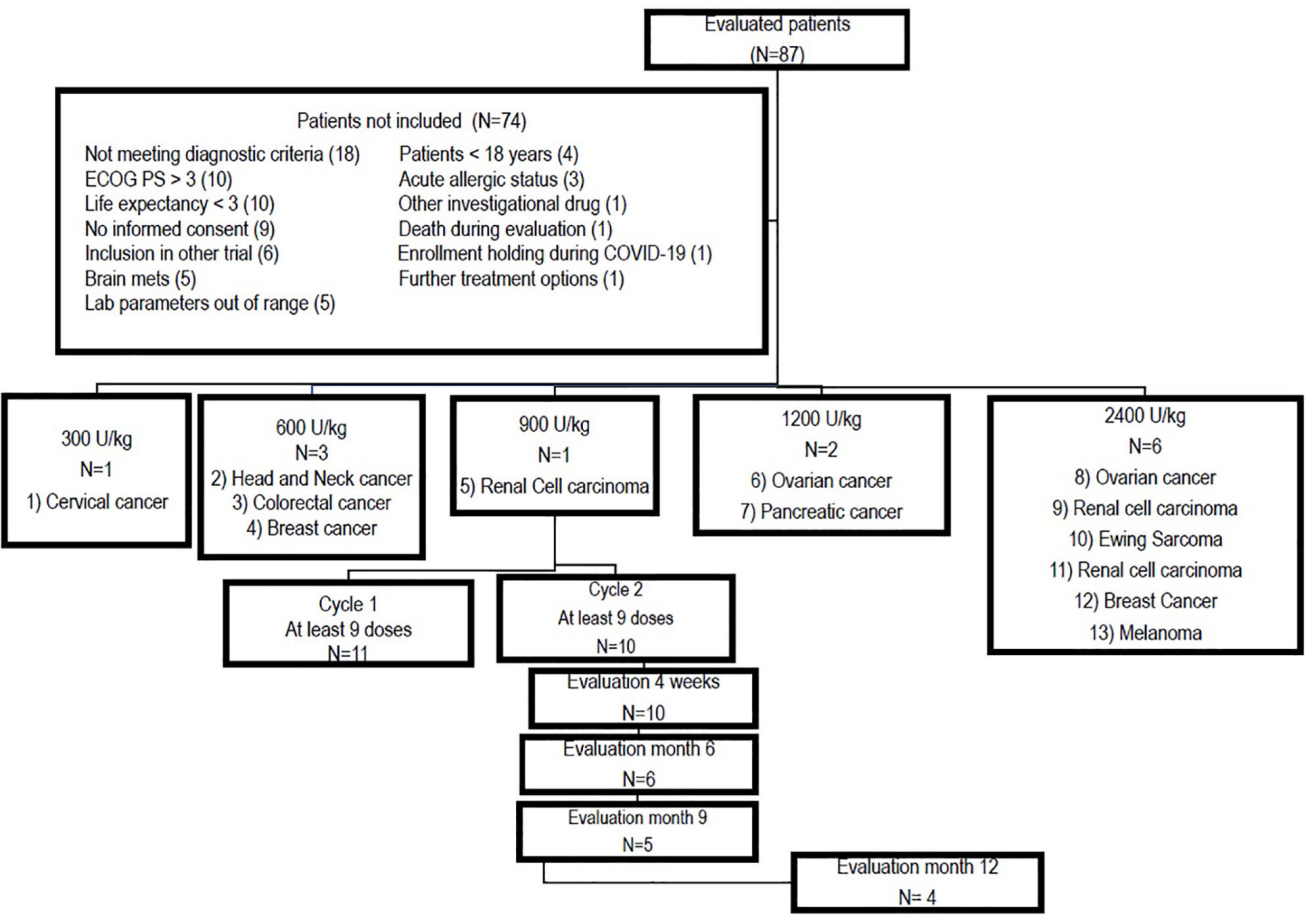
Consort diagram showing inclusion, treatment allocation per dose level and retention throughout the study.

Eighty-seven patients were evaluated for enrollment in the clinical trial, among whom, 13 individuals with advanced solid tumors with progressive disease and no further treatment alternatives were included between February 2020 and May 2023. **Figure 1** shows the consort diagram and explains the most important reasons for exclusion. The patients’ demographic characteristics are depicted in **Table 1**. Of the 13 subjects included in the study, 7 (58.3%) were male, and the age ranged between 25 and 69 years, with a median of 52 years. The tumor locations were as follows: cervical (1), head and neck (1), colorectal (1), breast (2), renal (3), ovarian (2), pancreatic (1), melanoma (1), and Ewing sarcoma. (1).

**Table 1:** Summary of patient demographics and baseline characteristics.

Demographics and baseline characteristics	Overall population (n=13)
Age (years) median (min, max)	52 (25-69)
Weight (Kg) median (min, max)	65 (47-85.5)
Sex	
Male (n/%)	6 (46.15%)
Female (n/%)	7 (53.84%)
Tumor localizations	
Cervical Cancer	1 (7.7%)
Head and Neck	1 (7.7%)
Colorectal cancer	1 (7.7%)
Breast Cancer	2 (15.4%)
Kidney Cancer	3 (23.1%)
Ovarian cancer	2 (15.4%)
Pancreatic adenocarcinoma	1 (7.7%)
Melanoma	1 (7.7%)
Sarcoma de Ewing	1 (7.7%)
Performance status	
ECOG 0	11 (84.6%)
ECOG 1	2 (15.4%)
Metastatic disease	
Yes	13 (100%)
Previous therapy	
Surgery	11 (84.6%)
Chemotherapy	12 (92.3%)
Radiation	8 (61.5%)
Immunotherapy	5 (38.5%)
Patients enrolled according dose levels	
300 IU/kg	1 (7.7%)
600 IU/kg	3 (23.1%)
900 IU/kg	1 (7.7%)
1200 IU/kg	2 (15.4%)
2400 IU/kg	6 (26.15%)

Enrollment was initiated at a dose of 300 IU/kg, and after the patient successfully completed the first cycle, the next subject was treated at a dose of 600 IU/kg. The first patient at this dose had serious adverse events, including severe left ventricular systolic dysfunction and pneumonitis. At the time of the adverse event, the patient had a respiratory infection that was not diagnosed at the baseline evaluation. Respiratory infection developed between the initial assessment and admission to the ICU. As stated in the protocol, two new individuals received a 600 IU/kg dose, with no evidence of new serious-related events. Then, escalation to 900 IU/kg was continued. No limiting toxicity was observed at 900 IU/kg, and enrollment was resumed at 1200 IU/kg. The first subject enrolled at the 1200 IU/kg level (patient 06) had an intercurrent illness that delayed the initiation of treatment with the no-alpha mutein. At that point, patient 07 was treated with the same dose to avoid treatment postponement. No serious adverse events were detected at a dose of 1200 IU/kg. In accordance with the research protocol, a higher dose was estimated to continue escalation, corresponding to the cohort of 2400 IU/kg, at which six patients were included. No evidence of DLT was found at a dose of 2400 IU/kg and the maximum tolerated dose was not reached. Overall, 11 individuals (86.4%) received the two treatment cycles established in the study, either completely (14 doses) or at least nine doses. Two patients (02 and 13) prematurely discontinued treatment and were classified as not evaluable (for objective response) per protocol. Patient 02 (600 IU/kg) discontinued treatment due to toxicity, while patient 13 voluntarily withdrew from the trial. Both patients received 3 doses of mutein.

All 13 patients who received at least one dose of treatment experienced adverse events. Two patients (02 and 05) experienced serious adverse events (SAEs). The serious events in patient 02 treated with 600 IU/kg consisted of ventricular systolic dysfunction and pneumonitis and were classified as possibly treatment-related. In contrast, patient 05 had a pneumothorax as a complication of central venous catheter insertion. Serious adverse events were classified as unrelated to the investigational drug.

Overall, 536 adverse events were detected, of which 469 (87.5%) were classified as related. The most frequent adverse events included chills (95), fever (73), tachycardia (51), nausea (46), vomiting (37), arterial hypertension (21), headache (17), and leukopenia (11). In terms of intensity, the most frequent events were mild (370 AEs, 69.02%), followed by moderate events (134 AEs, 25%). Furthermore, 28 severe (5.2%) and 4 very severe events (0.74%) were reported. Six patients (01, 02, 07, 10-12) showed severe toxicity. The frequency of the severe adverse events was as follows: hypertension (23.07%), systolic ventricular dysfunction, gamma glutamyl transpeptidase (GGT) increase, leukopenia, and lymphopenia (15.38%), followed by hypoxia, hypomagnesemia, pneumonitis, and pain (7.69%).

Four adverse events corresponding to lymphopenia episodes in 2 patients treated with a dose of 2400 IU/kg were classified as grade 4 according to the CTCAE table. Both patients started their first treatment cycle with normal lymphocyte count. On the second day, the counts decreased to values ​​lower than 0.20 x 10^9^/L. This lymphopenia had no clinical manifestations and lymphocyte counts recovered 24 h after the end of the cycle. No secondary infections occurred. The decrease in lymphocyte counts did not prevent the patients from receiving the second cycle as scheduled. Although these adverse events were classified as grade 4 considering the CTCAE grading, they were not life-threatening and did not require any urgent therapeutic intervention. Therefore, considering the severity algorithm defined in the protocol, these reactions were classified as non-serious. The team hypothesized that this transient lymphopenia was attributed to lymphocyte redistribution, i.e., lymphocyte migration into tissues, and not to activation-induced cell death, which would have led to a delayed recovery.

**Table 2** illustrates the frequency of serious and severe adverse events per dose level, while **Table 3** describes all the related adverse events that were detected in more than 20% of the treated individuals according the organ system and dose level.

**Table 2:** Safety according dose levels.

Adverse Events (AE) Summary	Assigned dose level											
	300 IU/kg		600 IU/kg		900 IU/kg		1200 IU/kg		2400 IU/kg		Total	
	No.	%	No.	%	No.	%	No.	%	No.	%	No	%
Number of patients with any AE	1	100	3	100	1	100	2	100	6	100	13	100
Number of patients with related AE	1	100	3	100	1	100	2	100	6	100	13	100
Number of patients with serious adverse events (SAE)	0	0.0	1	33.3	1	100	0	0.0	0	0.0	2	15.38
Number of patients with related SAE	0	0.0	1	33.3	0	0.0	0	0.0	0	0.0	1	7.69
Number of patients with severe AE	1	100	1	33.3	1	100	1	50.0	4	66.7	8	61.53
Number of patients with related severe adverse events	1	100	1	33.3	0	0.0	1	50.0	3	50.0	6	46.15

**Table 3:** Frequency of related adverse events detected in more than 20% of the patients according organ system and dose level. Number of adverse events are included in brackets.

Organ System	Related Adverse Event	Assigned dose level					
		300 U/kg (N=1)	600 U/kg (N=3)	900 U/kg (N=1)	1200 U/kg (N=2)	2400 U/kg (N=6)	Total Events/Pts (% of pts)
Heart disease	Sinus tachycardia	[5] 1 (100%)	[9] 3 (100%)	[1] 1 (100%)	[3] 2 (100%)	[32] 6 (100%)	[45] 13 (100%)
Diseases of the blood and lymphatic system	Anemia	[3] 1 (100%)	[1] 1 (33.3%)	–	[1] 1 (50%)	[2] 2 (33.3%)	[7] 5 (33.3%)
Diseases of the nervous system	Headache	[6] 1 (100%)	[4] 1 (33.3%)	[1] 1 (100%)	–	[1] 1 (16.7%)	[12] 4 (30.8%)
Gastrointestinal diseases	Nausea	[4] 1 (100%)	[5] 2 (66.7%)	–	[1] 1 (50%)	[32] 6 (100%)	[42] 10 (76.9%)
	Vomiting	[2] 1 (100%)	[3] 1 (33.3%)	–	[2] 1 (50%)	[28] 3 (50%)	[35] 6 (46.2%)
	Diarrhea	[1] 1 (100%)	–	–	–	[6] 2 (33.3%)	[7] 3 (23.1%)
	Abdominal pain	–	[1] 1 (33.3%)	–	–	[5] 2 (33.3%)	[6] 3 (23.1%)
General and administration site diseases	Chills	[16] 1 (100%)	[18] 3 (100%)	[6] 1 (100%)	[17] 2 (100%)	[37] 6 (100%)	[94] 13 (100%)
	Fever	[12] 1 (100%)	[28] 3 (100%)	[7] 1 (100%)	[10] 2 (100%)	[16] 5 (83.3%)	[61] 12 (92.3%)
Respiratory, thoracic and mediastinal diseases	Dyspnea	[1] 1 (100%)	–	[1] 1 (100%)	–	[3] 2 (33.3%)	[5] 4 (30.8%)
	Cough	[1] 1 (100%)	[2] 1 (33.3%)	–	–	[2] 1 (16.7%)	[5] 3 (23.1%)
Vascular diseases	Hypertension	[6] 1 (100%)	[1] 1 (33.3%)	–	[10] 1 (50%)	[4] 2 (33.3%)	[21] 5 (38.5%)
	Phlebitis	–	[3] 2 (66.7%)	[1] 1 (100%)	–	–	[4] 3 (23.1%)
Disorders of clinical laboratory parameters	Leukopenia	[4] 1 (100%)	–	–	–	[7] 2 (33.3%)	[11] 3 (23.1%)
	Lymphopenia	[2] 1 (100%)	–	–	–	[5] 2 (33.3%)	[7] 3 (23.1%)
	Increased aspartate aminotransferase	[1] 1 (100%)	–	–	–	[2] 2 (33.3%)	[3] 3 (23.1%)

**Figure 2:**
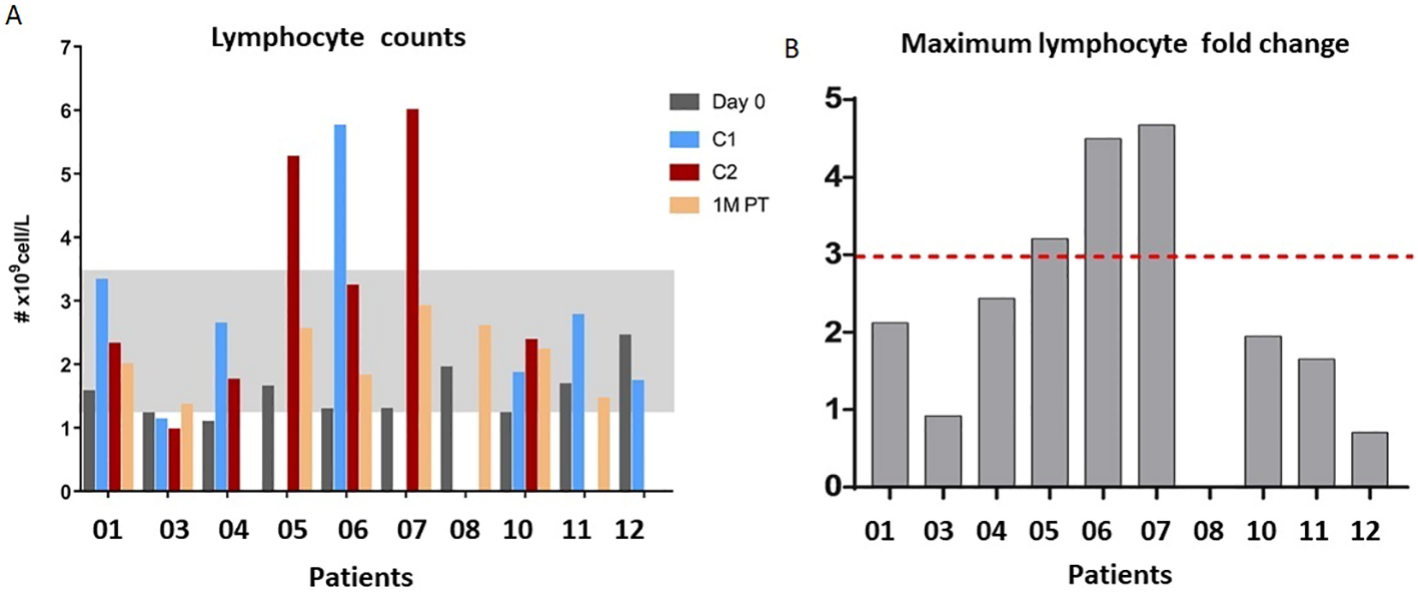
**(A)** Peripheral blood lymphocyte expansion after the treatment with no-alpha IL-2 mutein after cycle 1 (C1), cycle 2 (C2) or 1-month post-therapy (1M PT). **(B)** Maximum fold expansion per patient.

**Figure 3:**
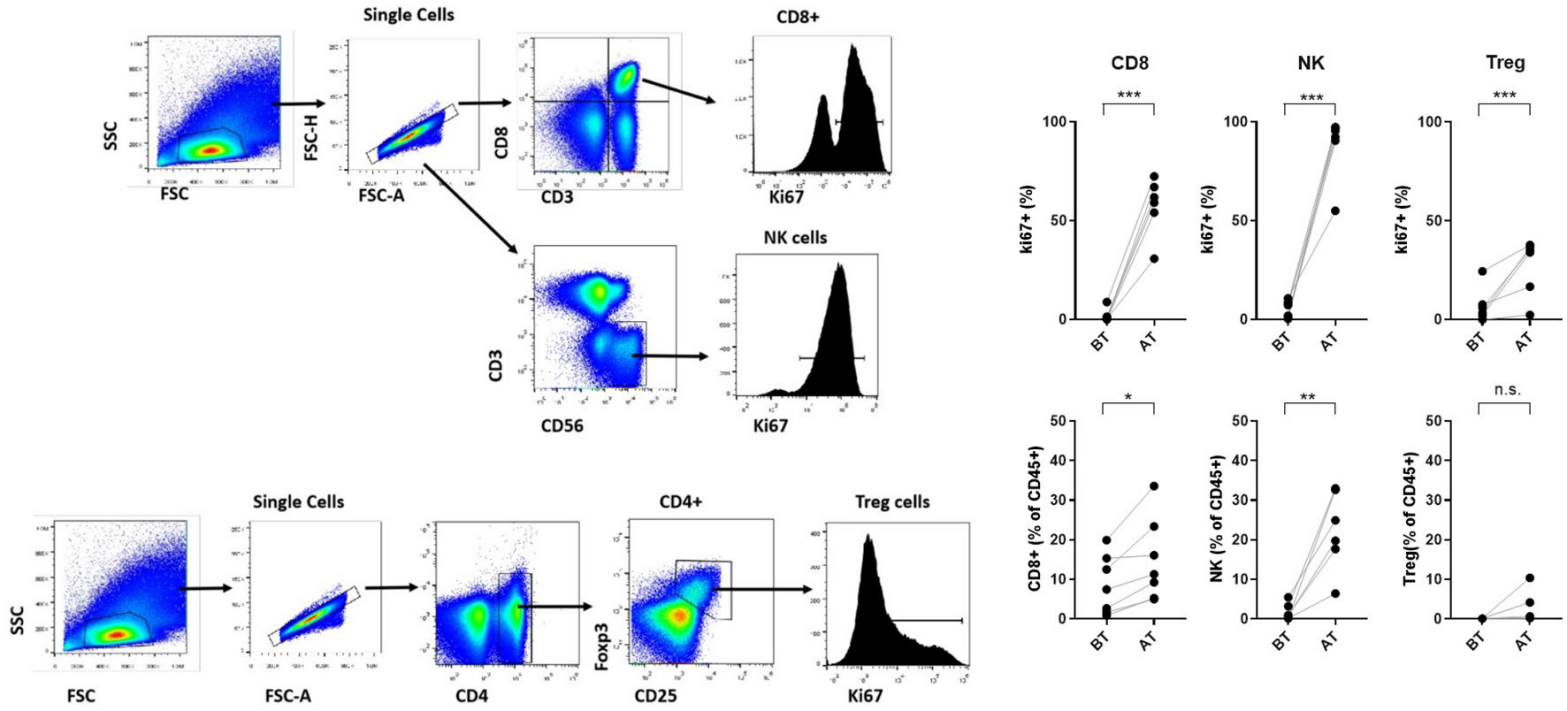
Gating strategy to analyze proliferation levels of NK, CD8 T cells and T-regs subsets. Proliferation levels and frequency of relevant lymphocyte populations (CD8 lymphocytes, NK and regulatory T cells) before and after treatment. Asterisks represent statistical significance where *** indicates greater significance.

Of the 13 patients enrolled in the study, 10 were eligible for pharmacodynamic evaluation. Blood samples were collected on day 0, 24 hours after each cycle, and 1 month after the end of treatment. Most patients experienced an increase in total peripheral blood lymphocyte count at one or more evaluation times, and in all cases, the levels returned to normal after 1 month. Contrary to what was expected, patients who received 900 and 1200 IU/kg doses (05-07) experienced the highest increases in the total lymphocyte count, reaching more than 3-fold expansion from baseline (**Figure 2**). The effect on the proliferation of relevant lymphocyte populations (CD8, NK, and T-regs) was assessed by measuring Ki67 levels. CD8 T lymphocytes and NK cells showed the highest proliferation, reaching an average of 60% and 87% of positive cells, respectively. T-regs proliferation also increased after treatment, but to a lesser extent, to an average of 27%. Furthermore, the increase in proliferation induced a proportional increase in the frequency of the referred lymphocytes, measured as the percentage of specific subpopulations among the CD45 cells. **Figure 2** illustrates the peripheral blood lymphocyte expansion after treatment with the non-alpha IL-2 mutein, while **Figure 3** shows the increase in the proliferation levels and the frequency of the relevant lymphocyte populations before and after treatment. All cell subtypes significantly increased their proliferation rate, but only CD8 T-cells and NK cells increased in frequency. The increase of T-regs was not significant.

**Figure 4:**
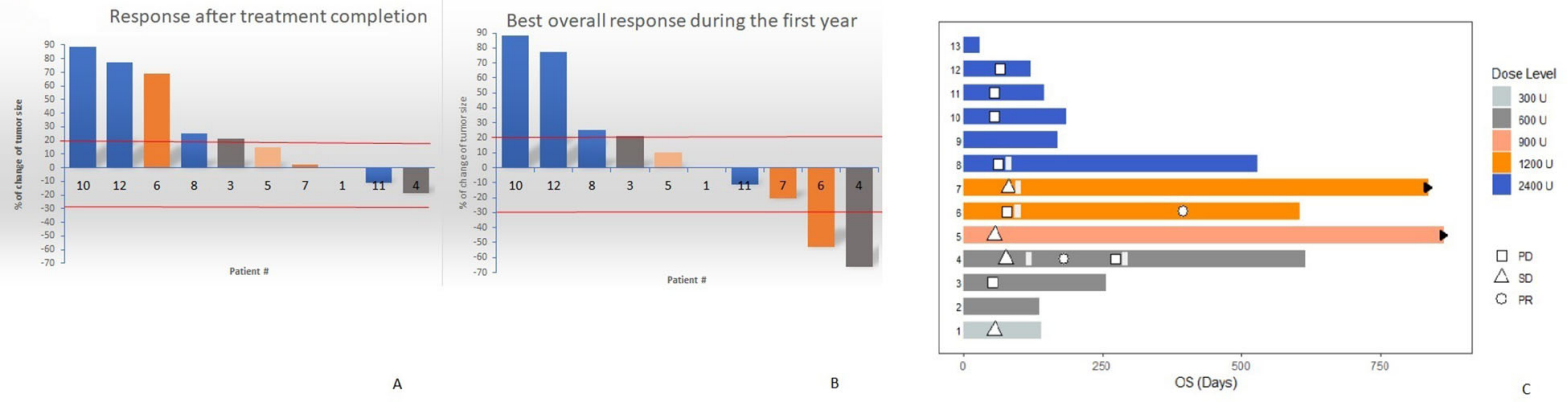
Water-fall plots showing the individual response according RECIST 1.1, after completing the mutein course **(A)** as well as the best overall response achieved during the 12 months follow-up **(B)**. **(C)** Swimmer plot describing the individual patient outcome including best response and survival. Patients 5 and 7 are alive at the moment of data cutoff (black arrows).

One month after treatment termination, the disease control rate (DCR) was 38.5% in the ITT setting (including all patients, regardless treatment compliance). However, 11 of the 13 patients were classified as evaluable considering treatment adherence and 10, had at least one response evaluation 4 weeks after completing the mutein course. Accordingly, in the per-protocol scenario, the DCR was 50%. Response evaluation continued for 1 year, at which, the DCR increased to 60%, including two partial responses and four stable diseases. **Figure 4** shows the individual response according to RECIST 1.1, after completing the mutein (panel A), as well as the best overall response achieved alongside the 12 months follow-up (panel B) in the 10 per-protocol evaluable patients. Each bar represents the change in tumor size from baseline. Only 4 patients received subsequent therapy after completing the mutein cycle.

During the first year of follow-up, partial responses were detected in two patients treated at doses of 600 IU/kg (patient 04) and 1200 IU/kg (patient 06). Patient 04 had a triple-negative breast cancer with metastases to the wall and soft tissue of the left hemithorax. Before enrollment in the study, she was treated with surgery, radiotherapy, and chemotherapy (four cycles of carboplatin + paclitaxel). The patient was administered mutein at a dose of 600 IU/kg to stabilize the disease. Two months after the last cycle, she started treatment with metronomic capecitabine (1 g) twice daily for 2 weeks, followed by a 1-week rest period, which was given as a 3-week cycle. At 6 months, the antitumor response was classified as partial. Three months later, the disease progressed, and the patient received radiotherapy to the sternum and left supraclavicular fossa. Her survival was 20.77 months. Patient 06 had an unresectable metastatic ovarian adenocarcinoma at diagnosis. Before inclusion in the mutein trial, she received neoadjuvant platinum-based chemotherapy, followed by surgery. Due to disease progression, the patient was classified as platinum-resistant and was enrolled in the trial. She was treated with a dose of 1200 IU/kg. The response at the end of the mutein course indicated disease progression. She received another line of chemotherapy (cisplatin and gemcitabine), achieving a partial response despite being platinum-resistant after the first exposure. The overall survival time of the patient was 19.47 months.

**Figure 4C** shows a swimmer plot describing the individual patient outcomes. Patients treated at intermediate doses (05; 900 IU/kg) and (06-07; 1200 IU/kg) had the largest survival. In addition to patient 06 (described above), patient 05, who had a renal cell carcinoma, has survived 28.8 months since inclusion in the trial, while patient 07, who had a metastatic pancreatic adenocarcinoma, has survived for 27.63 months, at the time of database closure. These two patients were alive at the time of writing this manuscript.

## Discussion

This manuscript describes the first in human evaluation of a novel interleukin 2 mutein, designed *in silico* to be less toxic and to preferentially activate immune effector cells.

IL-2 mutein was found to be safe, and dose-limiting toxicity was not observed. Dose escalation did not continue, as a greater clinical and pharmacodynamic effect was observed at intermediate doses.

Native IL-2 administration may cause death (2-4%) and capillary leak syndrome (CLS), characterized by extravasation of fluids and proteins (21). CLS may be associated with cardiac arrhythmias, angina, myocardial infarction, respiratory insufficiency requiring intubation, gastrointestinal bleeding, renal insufficiency, edema, and mental status changes (11, 12). The CLS might be attributed to the rupture of the pulmonary vasculature integrity through activation of endothelial cells expressing the trimeric IL-2 receptor (22). The wild-type cytokine can also provoke impaired neutrophil function and increased risk of disseminated infection, including sepsis and bacterial endocarditis (23). Regardless severity, the most frequent adverse events observed after using native IL-2 are hypotension, diarrhea, oliguria, chills, vomiting, dyspnea, rash, and bilirubinemia. In contrast, the most common toxicity observed after the no-alpha mutein treatment was chills, tachycardia, fever, and nausea.

In general, the adverse events seen with the mutein have been described for wild-type IL-2. Apart from the native, the mutated molecule did not induce capillary leak syndrome or death. The reduced interaction with the alpha subunit may explain its better safety profile.

However, all patients developed related adverse events that were mostly mild, while six individuals (46.5%) had severe related toxicity and only one patient (7.69%) showed serious events consisting of ventricular systolic dysfunction and pneumonitis. Although these SAEs were classified as possibly related, the patient also had an intercurrent respiratory infection, and the IL-2 mutein inflammatory response could have exacerbated the immunopathology. The presence of active infections was an exclusion criterion, which was presumably violated in this subject. A true causal relationship will only be established after treating a larger number of individuals. This patient discontinued the mutein administration, and fully recovered after receiving oxygen support, antibiotics, diuretics, low-dose heparin and an angiotensin-converting enzyme inhibitor. No further episodes of pneumonitis were seen in patients treated with the investigational drug, even at higher dose levels.

The non-alpha mutein increased lymphocyte counts. Notably, the effect was greater at intermediate doses (900 and 1200 IU/kg). At a dose of 1200 IU/kg, there was a 4-fold increase in absolute lymphocyte number. In addition, as predicted, IL-2 mutein preferentially expanded NK and CD8 T cells. A significant increase in the proliferation rate was observed in cytotoxic T lymphocytes and NK subpopulations. Treatment also produced a slight increase in the proliferation of regulatory T cells.

The non-linear pharmacodynamic effect of the mutein is not completely understood. It is worth noting that the pharmacodynamic effect of any IL-2 agonist might be complex provided it has both, stimulatory and regulatory effects, on the immune system (24). Overall, biological systems including cytokines often exhibit sigmoidal dose-response curves (25). IL-2, at low doses, preferentially expand T-regs; increasing concentrations activate effector T cells and NK cells and very high doses may induce activation-induced cell death (AICD) in effector T cells (25). A previous preclinical study on cancer vaccines genetically engineered to produce native IL-2, showed that optimal protection occurs at medium IL-2 levels, while lower levels reduce treatment success. On the contrary, high IL-2 levels completely impaired the generation of tumor-specific cytotoxic T lymphocytes and had no effect on tumors (26). In our dataset, the biggest clinical effect was seen at the intermediate doses of the non-alpha mutein.

In spite of their poor prognosis, the disease control rate was 50% for the evaluated patients who received at least 70% of the full treatment course. According the historical data, the response rate after using high doses of the wild type IL-2 was 15% in renal cell carcinoma patients (11) and 16% in metastatic melanoma (12).

Unexpectedly, two heavily pretreated patients achieved partial response after receiving subsequent therapy. Overall, it is not possible to determine with certainty whether the responses (or survival) were due to the additional therapies or whether treatment with the mutein influenced the new antitumor responses or the ultimate survival of each individual. Remarkably, subsequent treatment was always administered for palliative purposes, given that all patients had failed previous therapies for their disease, in accordance with national guidelines. Individual survival was higher at intermediate doses, coinciding with the greatest effect on immune effector populations.

Other modified IL-2 agonists have also been evaluated (13). The most advanced IL-2 variants in clinical settings are BEMPEG and nemvaleukin-alfa (27). NKTR-214 (Bempegaldesleukin/BEMPEG) is a pegylated IL-2. Considering the PEG chain insertion, BEMPEG did not recognize the CD25 subunit of the receptor. Despite the encouraging results of initial clinical trials, two large Phase III studies in metastatic melanoma or renal cell carcinoma failed to demonstrate efficacy. In a Phase III trial in 783 metastatic melanoma patients, the overall survival after the combined use of nivolumab plus BEMPEG did not differ from that of nivolumab alone. This combination was found to be more toxic. Immune-mediated adverse events occurred in 55% of the patients treated with the two medications (27). Regarding the specific effect on immune populations, BEMPEG and nivolumab preferentially increased the T-regs (8–10 fold) compared to the cytotoxic or NK counts, which only increased by 2–3 fold (28). These results contradict previous findings and may explain the failure of clinical trials. Other authors hypothesized that, *in vivo*, PEG could dissociate from BEMPEG and act as a conventional native cytokine and not as a non-alpha variant. The inability of effector cells to reach the tumor microenvironment has also been proposed as an explanation for the lack of efficacy (28).

In the second large clinical trial, 623 individuals with renal cell carcinoma were treated with BEMPEG plus nivolumab vs. sunitinib or cabozantinib. There were no differences in survival between the two groups, and more related adverse events were observed after the combination treatment (29).

Alternatively, ALKS-4230 (nemvaleukin-alfa) consists of a fusion protein where IL-2 binds to the external domain of the alpha chain of the receptor, intending to avoid interaction with the high-affinity complex (30). The results of a large Phase I/II trial (Artistry-I) have been reported. The trial included 243 patients with solid tumors, allocated to three cohorts, where nemvaleukin-alfa was used as monotherapy or in combination with pembrolizumab. The patients underwent repeated treatment cycles. Although the maximum tolerated dose was not reached, at the RP2D, 75% of patients had grade 3 and 4 reactions when receiving nemvaleukin-alfa alone. The most frequent toxicity was pyrexia, while the most common severe toxicities were neutropenia and anemia. Regarding efficacy, 65% of the patients had stable disease in the monotherapy cohort. The peak NK and CD8 T cell fold expansions were equivalent to 6.5 and 2.5 times. The authors claimed that T-regs had a minimal increase at RP2D (30). Even though these results are very promising, no results from controlled, randomized Phase III trials have been reported yet.

In summary, in this first in human trial in advanced cancer patients, the no-alpha mutein was safe and induced a preferential expansion of immune effector cells (NK and cytotoxic CD8 T cells) as compared to regulatory T cells. The disease control rate was encouraging for the heavily pretreated population. Once Phase I was completed, it was agreed to proceed to the Phase II trial at a RP2D of 1200 IU/kg. Considering the good safety profile, it was decided to administer the mutein in the conventional hospital ward and not in the ICU and to allow repeated treatment courses for those patients who do not progress and tolerate the medication. In agreement with the preliminary clinical effect, phase II will enroll patients in three 3 different cohorts: renal cell carcinoma or metastatic melanoma (cohort 1), castration-resistant ovarian cancer (cohort 2), and metastatic pancreatic cancer or triple negative breast cancer (cohort 3). The effect on immune subpopulations, pharmacokinetic parameters, safety and clinical endpoints will be further evaluated in the expansion cohorts. Upon completing the current Phase I/II study, new confirmatory controlled studies on safety, efficacy and biological effect will be designed.

## Data Availability

The raw data supporting the conclusions of this article will be made available by the authors, without undue reservation.
